# Supplementary figures and images for: Echocardiography overestimates LV mass in the elderly as compared to cardiac CT
Source: PLoS One. 2019 Oct 24;14(10):e0224104. doi: 10.1371/journal.pone.0224104 (PMC6812823; doi:10.1371/journal.pone.0224104)

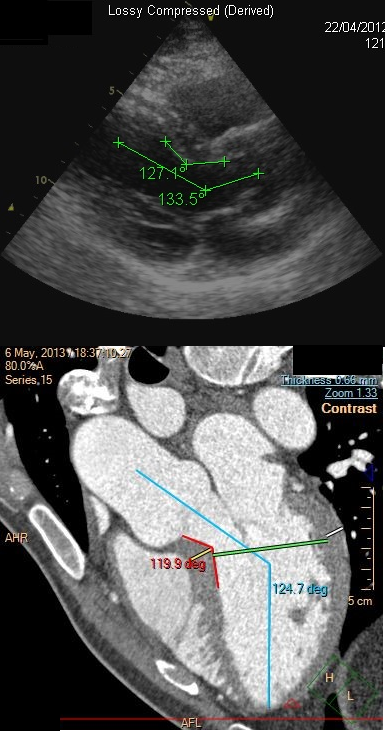

Supplement: S1 Fig — A. Echo Parasternal long axis view: Left ventricular outflow tract angulation (133.5) and aortoseptal angulation (127.1). B. CT three chamber view–left ventricular internal dimension (green), left ventricular outflow tract angle (blue), aortoseptal angle (red) (TIF) [file pone.0224104.s002.tif]

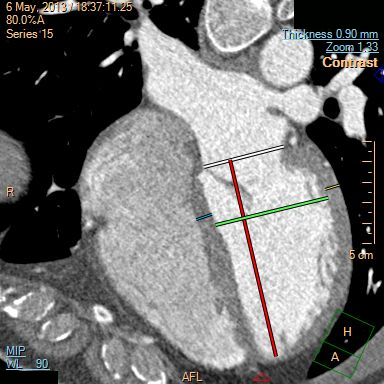

Supplement: S2 Fig — CT four chamber view–left ventricular internal dimension (green), septal wall thickness (blue), posterior wall thickness (yellow), LV length (red) (TIF) [file pone.0224104.s003.tif]
