# Supplementary material for: Echocardiography overestimates LV mass in the elderly as compared to cardiac CT
Source: PLoS One. 2019 Oct 24;14(10):e0224104. doi: 10.1371/journal.pone.0224104 (PMC6812823; doi:10.1371/journal.pone.0224104)
Supplement: Attachment — Submitted filename: response to reviewers.docx [file pone.0224104.s004.docx]

11 August 2019

Joerg Heber

Editor-in-Chief, PLOS ONE – Public Library of Science

Dear Prof. Heber:

We very much appreciate the opportunity to revise and resubmit our paper
“Echocardiography Overestimates LV Mass in the Elderly as Compared to Cardiac CT”

PONE-D-19-15733

to PLOS ONE. We have addressed the concerns of the academic editor and reviewers, as described below.

Academic Editor:

1. When submitting your revision, we need you to address these additional requirements.

Please ensure that your manuscript meets PLOS ONE's style requirements, including those for file naming.

*Authors’ response: We have edited the manuscript to meet PLOS ONE’s style requirements.*

2. In ethics statement in the manuscript and in the online submission form, please provide additional information about the patient records used in your retrospective study. Specifically, please ensure that you have discussed whether all data were fully anonymized before you accessed them and/or whether the IRB or ethics committee waived the requirement for informed consent. If patients provided informed written consent to have data from their medical records used in research, please include this information.

*Authors’ response: We have added the IRB ethics committee number and added the statement:*

“The ethics committee waived the requirement for informed consent.”

Response to Reviewers

Reviewer #1:

The main findings of this study is to correlate measurement of LV mass made by CT with traditional measurement made with echocardiography. Authors demonstrated that there is a good correlation with the 2 way of measurement but in elderly the echo systematically overestimate LV mass.
The main problem with the study is related to the methodological and related to the wrong way of measurement of LV septal wall thickness. As they report in figure 1 they both use proximal and and mid septum measurements and when using mid septum measurement much of the echo-CT discrepancy is eliminated. But this is just because using the proximal measurement in old patients with septal bulge is just uncorrected and the main finding is justified by the high prevalence of septal bulge in old patients. This is a main issue to be solved.

*Authors’ response: The authors agree with the reviewers that measurements performed according to present guidelines necessitate the inclusion of the septal bulge which leads to overestimation of LV mass. This study seeks to address precisely the concern raised by the reviewer.*

Minor Issues:
- the characteristics of the study population is not described. Are all patients hypertensive? what was the reason of the CT scan? what was the prevalence of hypertension or aortic valve disease in the two groups? Have you excluded patients with severe aortic stenosis?

*Authors’ response: As this was a retrospective study, the indications for imaging were not available to the investigators. Patients with severe aortic stenosis were included. The investigators believe that this broadens the spectrum of ventricular hypertrophy seen in the study, making the findings more robust and reflecting clinically relevant situations where estimation of LV mass is of importance.*

- in the results section, please report the median and IQR of the time between CT and Echo. It seems that in some patients the echo and CT have been within more than 3 months

*Authors’ response: The authors appreciate this concern. The manuscript was updated with the correct information in the first paragraph of the results section. The sentence in the results section now reads:*

“ Mean number of days between CT and echo studies was 3 with IQR of 34 days. “

-in the discussion section there is a long paragraph related to the fibrosis and increase cardiac collagen content. this is not pertinent to the this study.

*Authors’ response:The authors respect the reviewers comment. The two sentences discussing fibrosis, and myocardial stiffness were removed from the manuscript.*

Reviewer #2:

1) In general the tables should be reformatted to avoid character spillover between lines.

*Authors’ response: The tables have been updated so as to avoid character spillover between lines.*

2) Figures 3, 4 and 5, need to be reworked to make them more legible (larger font size), include measurement units on axis titles, and appropriate number of decimal places (Figure 3 in particular)

*Authors’ response: Figures 3,4, and 5 have been reworked with larger font sizes, measurement units and decimal places, as suggested by the reviewer.*

3) Throughout the document measurement units are absent in several places (e.g. LV mass in g) or erroneous (e.g. Table 1 RWT is unit-less, not cm)

*Authors’ response: The authors appreciate the reviewer’s comment and have placed the measurement units in the appropriate places in the document.*

4) Table 2 - should include LV mass data as this is the main metric discussed in the study.

*Authors’ response: The authors agree that the LV mass data is the main metric in the document. We have, however, placed this data in Table 3, as it permits a clearer and more informative presentation of the core findings of the study.*

5) There is mention of LV volumes briefly in the Discussion section, but these data are not shown. Consider adding the data to the tables and analysis if available or explaining its absence in more detail.

*Author’s response: The authors appreciate this comment as it is an important point. The data have been added to the table, and comments have been added in the results section. The addition reads:*

“There was no significant difference between LV volumes as calculated by echocardiography and CT.”

*and*

“No differences between age groups in either modality were seen in left ventricular internal diameter, volume or sphericity index.”

6) Readers may be interested in having measures LV mass correlation between modalities (for each cohort separately and combined). In addition, the linear regression function may be of interest as a method to calibrate between the two modalities. These data should be easily derived from the analysis in Figure 3.

*Authors’ response: This comment is well taken. We have incorporated the reviewer’s comment into the results section and Figure 3 which now reads:*

“Relative correlation coefficient between ASE and CT derived LV mass was 0.89; correlations by age group are presented in the scatter plot in figure 3. “

7) On the Bland-Altman plots - please add the p-value for mean difference (bias) different from 0? These are presented in the text and should correspond.

*Authors’ response: As the reviewer suggested ,this was incorporated into Figure 4.*

8) The text refers to Figure 4 as an appendix - I believe this is a typo.

*Authors’ response; This was a typo and has been corrected.*

9) Some readers may benefit of an explanation of why you analyzed only the septum and posterior wall thicknesses (i.e. why are other walls excluded).

*Authors’ response: The authors chose to use the measurements that are standardly obtained in echocardiography laboratories and are required for calculation of LV mass according to ASE and European Association of Cardiovascular Imaging. . We added this valid point to the study limitations.*

10) The discussion mentions several adjustments to echo LV mass measurements to improve agreement with CT and CMR, but falls short of making firm recommendations. Should echo LV mass be calibrated with an age adjusted function? should mid septum measurements be used? Or perhaps this data is too preliminary to make a firm recommendation?

*Authors’ response. The authors appreciate the reviewer’s acceptance of our findings. We believe that a larger follow up studies is needed to confirm these findings before making a firm recommendation for updating the guidelines to use mid-septal measurements.*

We appreciate the interest that you and the reviewers have taken in this manuscript.

Sincerely,

Donna R. Zwas, MD MPH
